# Barriers to accessing and engaging in healthcare as potential modifiers in the association between polyvictimization and mental health among Black transgender women

**DOI:** 10.1371/journal.pone.0269776

**Published:** 2022-06-16

**Authors:** Athena D. F. Sherman, Monique S. Balthazar, Gaea Daniel, Kalisha Bonds Johnson, Meredith Klepper, Kristen D. Clark, Glenda N. Baguso, Ethan Cicero, Kisha Allure, Whitney Wharton, Tonia Poteat

**Affiliations:** 1 Nell Hodgson Woodruff School of Nursing, Emory University, Atlanta, Georgia, United States of America; 2 Byrdine F. Lewis College of Nursing and Health Professions, Georgia State University, Atlanta, Georgia, United States of America; 3 Johns Hopkins School of Nursing, Baltimore, Maryland, United States of America; 4 Department of Community Health Systems, School of Nursing, University of California, San Francisco, California, United States of America; 5 Center for AIDS Prevention Studies, University of California, San Francisco, California, United States of America; 6 Casa Ruby, Washington, DC, United States of America; 7 University of North Carolina School of Medicine, Chapel Hill, North Carolina, United States of America; Oxford University Hospitals NHS Foundation Trust, UNITED KINGDOM

## Abstract

**Background:**

Black transgender women endure pervasive polyvictimization (experiencing multiple forms of violence throughout the lifespan). Polyvictimization is associated with poor mental health. Black transgender women also face barriers in access to healthcare, but the extent that such barriers modify the association between polyvictimization and poor mental health has not been described using convergent mixed-methods analysis.

**Methods:**

This convergent mixed-methods secondary analysis employs an intersectional lens and integrates two inter-related datasets to describe barriers to healthcare and the extent that such barriers modify the association between polyvictimization and mental health among Black transgender women. Investigators used survey data (n = 151 participants) and qualitative interview data (n = 19 participants) collected from Black transgender women (age 18 years and older) in Baltimore, MD and Washington, DC between 2016 and 2018. Analyses include thematic content analysis, bivariate analysis, joint display, and multivariate linear regression analysis examining mediation and moderation.

**Results:**

Joint display illuminated three domains to describe how barriers to healthcare present among Black transgender women–*Affordability*, *Accessibility*, and *Rapport and Continuity*. Independent t-tests revealed significantly higher polyvictimization, Post Traumatic Stress Disorder (PTSD), and depression scores among participants who reported at least one barrier to healthcare (BHI) compared to those who reported no barriers. BHI significantly moderated and partially mediated the association between polyvictimization and PTSD symptom severity and BHI fully mediated the association between polyvictimization and depressive symptom severity–when accounting for age and location.

**Discussion:**

Findings highlight the importance of access to healthcare in modifying the association between polyvictimization and PTSD and depression symptom severity among Black transgender women. Findings call for immediate interventions aimed at reducing barriers to healthcare and improved training for clinical providers serving Black transgender women.

## Introduction

Transgender and gender diverse (TGD) people (people whose gender differs from that normatively expected of their assigned sex at birth) experience high levels of stigma, including discrimination and victimization in community and healthcare settings, resulting in adverse mental health and widespread barriers to healthcare access and engagement [1–8]. Specifically, Black transgender women are disproportionately exposed to discrimination and victimization compared to cisgender peers (i.e., people whose gender aligns with that normatively expected of their assigned sex at birth) and White TGD peers [5, 9] and often experience multiple forms of victimization throughout their lifespan (i.e., polyvictimization) [10–12]. Such discrimination and victimization is associated with adverse mental health symptoms (i.e., symptoms of post-traumatic stress disorder [PTSD] and depression) among Black transgender women [10–14]. Black transgender women experiencing adverse mental health symptoms related to victimization, may be unable to access and engage in healthcare due to interpersonal and structural stigma-related barriers, such as denial of healthcare services or victimization and discrimination from providers [6, 8, 15]. Thus, barriers to accessing and engaging in healthcare may affect the ability of Black transgender women to obtain proper treatment and management of adverse mental health symptoms following exposure to violence.

Available data for TGD people indicate high rates of exposure to gender-based violence, sexual and physical violence, and discrimination [5, 16–18], with 58% of 27,715 participants in the United States Transgender Survey (USTS) reporting unequal treatment, verbal harassment, and/or physical attack for any reason in the past year [1]. The limited research on polyvictimization among TGD people reports gender-based disparities in victimization, with transgender girls and non-binary youth assigned male at birth (ages 14–19) reporting higher levels of polyvictimization (defined as experiencing more than 15 different types of victimization from the following domains: physical, bullying, sexual, child maltreatment, property victimization, and indirect victimization) than their TGD peers assigned female at birth and sexual minority cisgender peers (people whose gender aligns with that normatively expected of their assigned sex at birth who have a sexual orientation other than heterosexual) [4]. Polyvictimization has been directly associated with adverse mental health (i.e., symptoms of PTSD and depression) among TGD youth (ages 14–19) and Black and Latinx transgender women (adults), suggesting that the level of polyvictimization one experiences predicts the severity of adverse mental health symptoms [10–12]. Additionally, Black transgender women report nearly universal exposure to victimization in their lifetime, with Black transgender women in the US being murdered more often than TGD peers of any race or ethnicity [9, 18]. Black transgender women often attribute violent attacks to their gender expression or race [11] and those who are victims of crimes based on their race, gender or other biases, tend to have more severe mental health symptoms than those who experienced non-biased crimes [19].

There is a wealth of evidence stating that exposure to victimization [10, 11, 16, 20, 21] and barriers to healthcare access and engagement (e.g., financial concerns, transportation issues, lack of access to knowledgeable healthcare providers, and fears of mistreatment [6, 8, 22–24]) are associated with poor mental health and delays in treatment-seeking among TGD people [25, 26]. However, little research has examined the cumulative impact of barriers to healthcare and polyvictimization, on mental health symptom severity among TGD people [27]–and the experiences of Black transgender women are often underrepresented in the literature [25–29]. Existing literature suggests that TGD people, who previously had to teach their provider about TGD-related health, were four times as likely to have delayed healthcare seeking compared to those who did not have to teach their providers [1]. Specifically, Black transgender women report delayed treatment-seeking related to previous experiences of discrimination and victimization when seeking healthcare following a traumatic or violent event [11]. Conversely, experiencing healthcare encounters that were affirming of their gender, improved Black transgender women’s willingness to engage in care [30]. However, there are significant gaps in the literature regarding how cumulative barriers to healthcare access and engagement may affect the association between polyvictimization and mental health in this population [10–12]. Thus, more data are needed to increase our understanding of (a) how barriers to accessing and engaging in healthcare present for Black transgender women and (b) determine if barriers to healthcare modify the association between victimization and mental health symptom severity.

### Purpose

To address gaps in the literature, this convergent mixed-methods study [31] employs an intersectional lens, integrating two inter-related datasets to (Aim 1) describe the characteristics (thematically explore the most impactful barriers to accessing and engaging in healthcare, as reported by community members), prevalence, and correlates of barriers to healthcare access and (Aim 2) examine whether barriers to accessing healthcare modify the association between polyvictimization and PTSD symptom severity and depressive symptom severity (*hypothesis*: barriers to healthcare access would be associated with increased severity of symptoms of depression and PTSD for those with increased polyvictimization) among Black transgender women in Baltimore, MD and Washington, DC.

## Methods

### Study design and setting

This study uses quantitative data from the study Supporting Transgender Research and Opportunities in the Baltimore/DC Environment (STROBE) [10, 32, 33] and qualitative data from the study TransConnect: A mixed methods study of pathways to health for Black and Latinx transgender women who have survived violence (TransConnect) [11]. The purpose of the STROBE study was to “explore, quantify, and develop a response to the burden of Human Immunodeficiency Virus (HIV) affecting Black and Latinx transgender women in Baltimore, MD, and Washington, DC” (p. 359) [10]. The TransConnect study (2019), among Black transgender women in Baltimore, MD, and Washington, DC, was designed to expand upon and explain findings of the STROBE study (2017–2018) related to polyvictimization, using an explanatory mixed-methods design to explore the help-seeking process post-exposure to violence [11, 34]. The TransConnect study and the STROBE study received approval from the Johns Hopkins Institutional Review Board. Participants consented verbally in person and no signatures were obtained to maintain anonymity. The data from both studies cannot be shared publicly because participants did not agree to sharing of data outside of the research team or institution without approval. However, data are available from the Johns Hopkins Institutional Data Access / Ethics Committee (contact via jhmeirb@jhmi.edu) for researchers who meet the criteria for access to confidential data. Details about funding for the original TransConnect and STROBE studies can be found elsewhere [2, 10, 11].

#### STROBE methods

The STROBE study collected quantitative survey data from Black and Latinx transgender women from Baltimore, MD and Washington, DC metro areas [10, 32, 33]. Participants completed an in-person researcher- and computer-assisted survey (lasting an hour on average), capturing data on various topics, such as demographics, mental health, social support, HIV status, and substance use, among other topics [10, 32, 33]. Detailed descriptions of the original study aims, recruitment strategies, inclusion and exclusion criteria, materials development, data collection, and community advisory board (CAB) guidance for STROBE can be found elsewhere [32, 33].

#### TransConnect methods

The TransConnect study collected mixed-methods data from Black transgender women from Baltimore, MD and Washington, DC metro areas [11]. TransConnect participants completed individual semi-structured interviews and a short survey (lasting roughly 2 hours) capturing data including victimization history and demographics. During the qualitative interviews, participants were asked what they perceived as the barrier that had the greatest impact on their ability to access and engage in healthcare. Detailed descriptions of the study aims, recruitment strategies, inclusion and exclusion criteria, materials development, data collection, and community advisory board guidance for TransConnect can be found elsewhere [11].

#### Participants

For this analysis, only those who endorsed Black or African American (by itself or in addition to other race or ethnic selections), endorsed Female/woman, Transgender female or trans woman, and who reported their sex assignment at birth as male were included in this analysis. From this point forward, STROBE participants (n = 151) will be referred to as ‘quantitative participants’ (using survey data only) and TransConnect participants (n = 19) will be referred to as ‘qualitative participants’ (using qualitative interview data and demographic data only). Individual participants will be referred to by pseudonyms of precious stones (e.g., Ruby, Sunstone, etc.).

#### Variables

Several demographic variables were captured in the quantitative survey (i.e., age, gender, sex, sexual identity, location, race, ethnicity, employment, education, United States (US) immigration status, and US citizenship status). Race and ethnicity were identified by a ‘select all that apply’ item. The Polyvictimization Inventory (PVI; 15-items; binary yes/no; range 0 to 15 with higher scores denoting more exposure to different types of violence from three domains including physical violence, sexual violence, and threats of violence; Cronbach’s α of current study = .91) was originally created for the STROBE study [32, 33] and captures the lifetime exposure to violence. Researchers have examined polyvictimization using various cut offs (1) to identify ‘poly-victims’ or (2) as a continuous variable to examine the condition of being polyvictimized [27]. Both approaches to measuring polyvictimization have been associated with adverse mental health symptoms among TGD people [10, 12, 27]. For the purposes of our secondary data analysis PVI was operationalized as a continuous variable to capture the severity of polyvictimization instead of the prevalence of polyvictimization using a cut-off value [27]. Items resemble “Has anyone slapped you or thrown something at you that could hurt you?” The Patient Health Questionnaire 2 clinical screener (PHQ2; 2-item; 4-point Likert scale; range 0 to 6) captures self-reported symptoms of depression over the past 2-weeks and was operationalized as a continuous variable for our secondary data analysis (Cronbach’s α of current study = .68) [35]. Higher scores are representative of more severe depressive symptoms. Items resemble “Over the last 2 weeks, how often have you been bothered by little interest or pleasure in doing things?” The Primary Care PTSD clinical screener (PC-PTSD; 4-items; binary yes/no; range 0 to 4) was designed for use in primary care settings to capture self-reported symptoms of PTSD (i.e., re-experiencing, numbing, hyperarousal and avoidance) in the past 30 days and was operationalized as a continuous variable (Cronbach’s α of current study = .78) [36]. Higher scores are representative of more severe PTSD symptoms. Items resemble “In your life, have you ever had any experience that was so frightening, horrible, or upsetting that, in the past month, you: Have had nightmares about it or thought about it when you did not want to?” [36]. The Barriers to Accessing Healthcare Inventory (BHI) was created for the STROBE survey to identify self-reported barriers to healthcare access [32, 33]. Specifically, STROBE participants were asked, “What are some of the challenges you face when accessing healthcare? Please say yes or no to the following: (1) Time; (2) Transportation; (3) Worried about safety getting to/from the health provider; (4) Child care; (5) Cost; (6) No health coverage (i.e., no health insurance); (7) Hours not convenient; (8) Mistreatment by healthcare staff and/or other patients for being transgender; (9) You have had bad experiences in the past; (10) You feel like healthcare providers are not comfortable caring for transgender patients; and (11) Other [Fill In].” For bivariate and multivariate analysis, we removed the items “Child care” (*n =* 1) and “other” (*n =* 4) from the BHI cumulative continuous scoring because the equal exposure opportunity of the items was unclear. However, both items are described in detail in the results. The final BHI used for our secondary data analysis include 9 of 11 items (binary yes/no; range 0 to 9; Cronbach’s α of current study = .70) and was operationalized as a binary variable (i.e., had at least one barrier vs. no barriers reported) for bivariate analyses and a continuous count variable for multivariate analyses in order to examine the effect of experiencing cumulative multiple barriers.

### Data analyses

To determine the appropriateness of mixing the two inter-related datasets, a comparison of demographic variables was completed, which revealed no significant differences between the samples based on age (*t*(168) = .30; *p* = .67) or education (X^2^(6) = 5.95; *p =* .43; see Table 1). Data for employment and citizenship were not compared between the two samples due to data missingness. Based on dataset comparison and participant overlap (36.8% of TransConnect participants confirmed that they also completed the STROBE survey), it was determined that dataset integration was appropriate to address the aforementioned mixed-methods aims of this analysis. Thus, the current analysis uses a convergent mixed-methods design to compare findings of the two existing datasets using joint display to guide the discussion. Convergent mixed-methods research integrates, or merges, a quantitative and a qualitative dataset to examine an appropriate research question [31, 34]. This approach provides greater contextualization and a more comprehensive understanding of a phenomenon than can be described by using either quantitative or qualitative analysis separately [31, 34].

**Table 1:** Demographics among Black transgender women among included STROBE Participants (N = 151) and included TransConnect participants (N = 19).

	STROBE				TransConnect	
	No Barriers (*n* = 35)	Barriers (*n* = 116)	Total (*N* = 151)		Total (*N* = 19)	
	*M*(*SD*)	*M*(*SD*)	*M*(*SD*)	Barriers v. Non-barriers (*p)*	*M*(*SD*)	STROBE v. TransConnect (*p)*
**Age**	42.0(12.3)	39.4(12.8)	40(12.7)	.28 ^a^	39.1(13)	.67 ^a^
	***n*(%)**	***n*(%)**	***n*(%)**	***p***	***n*(%)**	
**Completed STROBE Survey**	35(100)	116(100)	151(100)		7(36.8)	-
**Location**				.11 ^*x*^		.06 ^*x*^
DC	19(54.3)	80(69)	99(65.6)		10(52.6)	
Baltimore	16(45.7)	36(31)	52(34.4)		9(47.3)	
**Hispanic/Latinx**				1.00 ^*f*^		.55 ^*x*^
Yes	2(5.7)	9(7.8)	11(7.3)		1(5.3)	
No	33(94.3)	107(92.2)	140(92.7)		18(94.7)	
**US Citizen**				.09 ^*x*^		-
Yes	21(60)	87(75.7)	108(71.5)		19(100)	
No	0	0	0		0	
Missing	14(40)	29(25.2)	43(28.5)		0	
**Employment**				.17 ^+^		-
Unemployed	16(45.7)	63(54.3)	79(52.3)		3(15.8)++	
Student/Retired/Disability/Other	5(14.3)	23(19.8)	28(18.5)		10(52.6)++	
Employed	14(40)	30(25.9)	44(29.1)		11(57.9)++	
**Education**				.09 ^+^		.43 ^*x*^
Did not complete High School	8(22.9)	28(24.1)	36(23.8)		7(36.8)	
High School diploma or GED	6(17.1)	43(37.1)	49(32.5)		6(31.6)	
Technical/Vocation School	4(11.4)	10(8.6)	14(9.3)		2(10.5)	
Some College/Associate degree	15(42.9)	31(26.7)	46(30.5)		2(10.5)	
Completed college/ Bachelor’s Degree	1(2.9)	1(0.9)	2(1.3)		1(5.3)	
Graduate School	0(0)	3(2.6)	3(2)		1(5.3)	
Missing	1(2.9)	0(0)	1(0.7)		0	

**Note.** f (Fisher’s Test); + (Likelihood Ratio); ++ (do not equal 100% because participants were allowed to select all that apply); M (mean); Max (maximum score); Min (minimum score); x (Pearson Chi Squared); SD (standard deviation); a (t-test); all participants were assigned male sex at birth.

#### Quantitative analyses

The distributions of variables were examined for outliers, missing data, and normality. Statistical analyses were conducted by the first author using SPSS, Version 25 [37]. Survey data (*N* = 151) were analyzed using a correlation matrix, Student’s t-tests, and multiple linear regression modeling with interaction terms (i.e., the interaction between polyvictimization and barriers to healthcare [PVI X BHI]). Demographic items (age and location) included in the models were determined based on previous literature [10]. Separate models were created based on the outcome variables (symptoms of PTSD and symptoms of depression) with predictors of PVI, BHI, their interaction (i.e., PVI X BHI), and age and location. Models were fit separately to account for the BHI continuous variables and the coinciding interaction terms. Collinearity was assessed, standardized and unstandardized regression coefficients were compared, and significance testing was done at an alpha level 0.05 (two-sided).

A simple mediation analysis was performed using PROCESS: A Versatile Computational Tool for Observed Variable Mediation, Moderation, and Conditional Process Modeling for SPSS (bootstrapping = 5000) [38]. The outcome variables for analysis (in separate models) were A) PTSD symptom severity and B) depressive symptom severity. The predictor variable for the analysis was polyvictimization (PVI). The mediator variable for the analysis was BHI. Possible confounders of age and location were accounted for in the models.

#### Sensitivity analyses

All sensitivity analyses were conducting using G*Power software [39]. Post hoc analysis of power for examining moderation in a linear regression using an f test determined that given the sample size of 151, a power of 80%, an alpha level of .05, and four predictors, we can detect an effect size of f^2^ = 0.08 to be statistically significant, which is considered a small to medium effect size [40–42]. Post hoc analysis of power for examining mediation in a linear regression using an f test determined that given the sample size of 151, a power of 80%, an alpha level of .05, and four predictors, we can detect an effect size of f^2^ = 0.05 to be statistically significant, which is considered to be a small to medium effect size 40–42].

#### Qualitative analyses

TransConnect researchers transcribed audio-recorded interviews verbatim and processed the transcripts using the core tenets of narrative analysis, explained in detail elsewhere [11]. For this analysis, processed transcripts were uploaded into Dedoose mixed-methods software for thematic content analysis with tenets of narrative analysis [43–46]. Each transcript was coded independently in Dedoose by two co-authors and conflicts were resolved by one additional co-author. Codes were compared and collapsed to create themes by the first and third authors. Excerpts were chosen to represent the identified themes and were integrated into the results. Analyses were guided by the Gender Minority Stress Model [47]. All participants were assigned the name of a precious stone as a pseudonym to protect their identities.

The research team for the current analysis consisted of an African American heterosexual transgender woman, an African American lesbian cisgender woman, two African American heterosexual cisgender women, a Caribbean American heterosexual cisgender woman, an Asian American heterosexual cisgender woman, a White queer non-binary person, a White heterosexual transgender man, a White gender fluid pansexual person, a White pansexual cisgender woman, and a White lesbian cisgender woman. Researchers who completed thematic coding also completed bracketing via reflexive journaling to identify potential biases and establish dependability [48]. Researcher journal entries were reviewed as a team and included the following main themes: (a) participants would report high exposures to violence, sex work, homelessness, and discrimination and (b) participants would endorse high rates of barriers to healthcare. These themes were considered, and their influences discussed throughout data analysis. To establish credibility of the findings, themes describing the most impactful barriers to care were discussed with the TransConnect Community Advisory Board (CAB) members to facilitate a form of member checking. Triangulation of multiple sources of data (i.e., field notes, memoing, input from CAB members, quantitative survey data, and interview transcripts) allowed for increased confirmability of the findings.

#### Integration of findings

Convergent mixed-method design was used to integrate data and address the aforementioned aims. The existing quantitative measure of barriers to healthcare access used in the STROBE study was created based on findings from generalized TGD and cisgender research–not specifically created for or based on the experiences of Black transgender women [33]. Thus, findings, which qualitatively explored the most impactful barriers to accessing and engaging in healthcare (as reported by community members), were integrated with quantitative findings to determine how barriers to healthcare present among Black transgender women. Specifically, thematic qualitative findings were compared with the quantitative findings to identify where integration enhanced explanation and were then reviewed with the TransConnect CAB for accuracy, appropriateness, and completeness [31, 34]. CAB members emphasized how one’s social disadvantage and ability to complete procedural tasks necessary for acquiring healthcare can contribute to one’s overall perceived access and engagement in healthcare, which was added and considered in the final discussion of the results. Findings were then structured, presented, and discussed using convergent mixed-methods joint display [31].

## Results

### Participants

A total of 154 Black transgender women completed the STROBE quantitative survey; however, three were missing data related to symptoms of PTSD or depression (less than 5%) and were removed from the current analysis using listwise deletion, bringing the sample size to 151. Nineteen Black transgender women completed the TransConnect qualitative interviews. Table 1 describes quantitative participant demographic information stratified by those who reported at least one barrier to healthcare compared to those who reported none in the quantitative sample (no significant differences were found between groups) and demographic information for qualitative participants included in this analysis. All participants included in this analysis were 18 years old or older [quantitative mean [*M*] (standard deviation [*SD*]) = 40(12.7), range = 19–82; qualitative *M*(*SD*) = 39.1(13.0), range = 23–60]. Age and location were significantly correlated with a younger sample residing in Baltimore, MD (*M*(*SD*) = 36.7(11.9) years old) versus Washington, DC (*M*(*SD*) = 41.7(12.8) years old).

### The characteristics, prevalence, and correlates of barriers to healthcare access among Black transgender women

Nearly 77% (*n* = 116) of quantitative participants reported at least one barrier to accessing healthcare, via the BHI (mean [*M*] *=* 2.5; standard deviation [*SD*] = 2.1; range 0–8). The most commonly endorsed barriers were transportation (*n* = 74; 49%), time (*n* = 58; 38%), and cost (*n* = 54; 36%) (Table 2). Bivariate analyses revealed significant correlations between barriers to healthcare (BHI), polyvictimization (PVI), Patient Health Questionnaire 2 scores (PHQ2), and Primary Care PTSD (PC-PTSD) scores (Table 3). Further investigation via independent t-tests revealed significantly higher PVI, PHQ2, and PC-PTSD scores among participants who reported at least one barrier to healthcare compared to those who reported no barriers (Table 4). Average scores among the complete sample were 7.7 (*SD* = 4.8) for PVI, 1.8 (*SD* = 1.7) for PHQ2, and 1.7 (SD = 1.5) for PC-PTSD. The PHQ2 and PC-PTSD scores were significantly correlated; however, item content review of the measures revealed no specific symptom overlap that may have driven the association (i.e., no two items assessed the same symptom when comparing both measures). Lastly, Baltimore, MD based participants had significantly lower PVI (*M*(*SD*) = 6.1(4.6)), PHQ2 (*M*(*SD*) = 1.4(1.2)), and PTSD-PC (*M*(*SD*) = 1.3(1.4)) scores than Washington, DC participants; PVI (*M*(*SD*) = 8.6(4.7)), PHQ2 (*M*(*SD*) = 2.0(1.9)), and PTSD-PC (*M*(*SD*) = 1.9(1.5)).

**Table 2:** Barriers to accessing and engaging in healthcare: Joint display of quantitative and qualitative data.

Barriers to Accessing Healthcare (N = 151)				Most Impactful Barriers to Accessing and Engaging in Care (N = 19)				
	BHI Barriers	*n*	%	Themes	Definition	*n*	%	*Type*
**Affordability**	Cost	54	36	Financial strain/Cost	Inability to afford healthcare, obtain health insurance, or have procedures covered by health insurance when insured.	3	16	Access & Engagement
	No health insurance coverage	22	15					
**Accessibility**	Transportation	74	49	Limited number of gender-affirming providers	A shortage of gender-affirming and knowledgeable healthcare providers. Gender-affirming care is the delivery of culturally competent and inclusive care to TGD people in the context of primary and specialty healthcare and provision of gender-affirming medical interventions.	6	32	Access
	Time	58	38					
	Hours not convenient	35	23					
	Worried about safety getting to and from the healthcare provider	35	23					
	*Other	4	3					
	*a*. *“Restrictions and coding in terms of insurance”*							
	*b*. *“Waiting list for providers experienced with trans populations” *							
	**c. *“Barriers to getting credentials and be identified/intake to gain linkage to services*, i.e. *medical insurance; gawking individuals in the lobby/reception area*, *even at [an local LGBTQ+ clinic]” *							
	*d*. *“Haven’t updated medical records system to record gender identity”*							
	*Child care	1	1					
**Rapport and Continuity**	Previous bad experiences	42	28	Stigma and mistreatment by healthcare staff	Anticipated stigma and experiences of interpersonal stigma (perpetrated from one person to another, often described as discrimination) and mistreatment by healthcare professionals.	6	32	Access & Engagement
	Feel like healthcare providers are not comfortable caring for Transgender patients	38	25	Misalignment of provider and patient perception of care	Patients did not agree with the providers opinions of health-related topics or treatment approach.	5	26	Engagement
	Mistreatment by healthcare staff and/or other patients for being transgender	21	14	Complex social and structural vulnerability	Variables present in the participants life that act as a barrier, such as food and housing insecurity, lack of adequate income, substance use, and neighborhood disorder.	4		Access & Engagement
				Continuity of care	Lack of a consistent provider from one visit to the next.	3	16	Engagement

Note.

*not included in quantitative analysis

**this barrier aligns with Affordability, Accessibility, and Rapport and Continuity. Participants were able to ’select all that apply’ for barriers to access in the survey and were able to qualitatively identify more than one most impactful barrier in the interviews. Thus, percentages will not add up to 100%.

**Table 3:** Pearson correlations for quantitative sample (N = 151).

	Age	Polyvictimization	Depression	PTSD	Healthcare Barriers	Location^+^
**Age**	1					
**Polyvictimization**	-0.01	1				
**Depression**	-0.07	0.25**	1			
**PTSD**	-0.13	0.40**	0.46**	1		
**Healthcare Barriers**	-0.09	0.33**	0.31**	0.44**	1	
**Location** ^**+**^	-.19*	-0.24**	-0.19*	-0.17*	-0.11	1

Note

*(p< 0.05; 2-tailed)

**(p< 0.01; 2-tailed); + (0 = Washington, DC and 1 = Baltimore, MD); Depression (Patient Health Questionnaire 2 clinical screener); Healthcare Barriers (Barriers to Healthcare Inventory); PTSD (Primary Care PTSD clinical screener); Polyvictimization (Polyvictimization Inventory).

**Table 4:** Independent T-tests of key variables by exposure to healthcare barriers among the quantitative sample (N = 151).

	No Barriers (*n* = 35)	Barriers (*n* = 116)		
	*M*(*SD*)	*M*(*SD*)	*t*	*MD* (*Std*. *Error Diff*.)
**Polyvictimization**	5.4(4.8)	8.4(4.6)	*t*(149) = -3.4	-3.0(0.9)**
**Depression**	1.1(1.3)	2(1.8)	*t*(149) = -2.8	-0.9(0.3)**
**PTSD**	0.6(1.1)	2.0(1.5)	*t*(73.7) = -6	-1.4(0.2)**

Note

**(p< 0.01; 2-tailed); Depression (Patient Health Questionnaire 2 clinical screener); M (mean); Max (maximum); MD (mean difference); Min (minimum); PTSD (Primary Care PTSD clinical screener); Polyvictimization (Polyvictimization Inventory); SD (standard deviation).

During the qualitative interviews, participants were asked what they perceived as the barrier that had the greatest impact on their ability to access and engage in healthcare (referred to as a “primary barrier”). Nearly 84% (*n* = 16) of qualitative participants reported at least one primary barrier to accessing or engaging in healthcare (*M*(*SD*) *=* 1.8(2.3); range 0–8). Overall, the most impactful barriers to care reported by our study participants were categorized into three overarching domains, **Affordability, Accessibility,** and **Rapport and Continuity.**

### Affordability

Affordability refers to one’s ability to afford health care costs without financial hardship and encompasses our theme of *Financial Strain/Cost* [49] (see Table 2 for theme definition) and corresponds with BHI items: *Cost* and *No health insurance coverage*.

#### Financial strain/cost

Participants (*n* = 3 of 19 participants with 5 total endorsements) felt financial strain/cost was one of the most impactful barriers to accessing and engaging in healthcare. The participants strongly desired care; however, the lack of adequate health insurance related to joblessness, dependency on government health insurance, or charity-assisted payments that did not always cover healthcare expenses, made it difficult to afford healthcare. In these cases, participants would sacrifice food and other necessities to save money; some even resorted to participating in illegal actions, such as theft and fraud, to access or maintain care. Ultimately, these efforts often failed since the illegal activities were not sustainable and many times ended in incarceration. Attaining financial assistance to cover healthcare was complicated and exhausting for some participants. Amethyst shared:

*I did all my homework, and it took me from February until May to get an appointment. I did everything…I did the letters, everything you ’posed to do…So the lady called me she said, ‘I’m from the payment department.’ She said, ‘Well, we don’t take DC Medicaid*.’ *My understand[ing] was, if my main doctor was getting all this together for me, you should have gotten that part together for me with the insurance. Because my Medicare pays 80%, and DC pays 20%. So, they were saying, ‘Well, you have to pay $2400.’ I didn’t have no $2400… they just said, ‘…we’re just not gonna do the surgery.’ So, there’s nothing else I can do… I would have done that, if I had to give them just $5 a month [a payment plan]*.

Even when participants thought they had completed all the steps to receive financial assistance to cover healthcare cost and were granted permission to proceed with care, a problem would arise and participants still struggled to receive care, as seen with Amethyst.

### Accessibility

Accessibility refers to the availability of good quality health services in terms of distance, transportation, operating hours, appointment availability, etc. allowing people to obtain needed services in a timely manner [49]. Specifically, Accessibility encompasses our theme of *Limited Number of Gender-affirming Providers* (see Table 2 for theme definition) and corresponds to BHI items: *Transportation*, *Time*, *Hours not convenient*, *Worried about safety getting to and from the healthcare provider*, *and Child Care*.

#### Limited number of gender-affirming providers

Gender-affirming care is the delivery of culturally competent and inclusive care to TGD people in the context of primary and specialty healthcare and provision of gender-affirming medical interventions (interventions aimed at aligning one’s body with their gender identity or reducing gender dysphoria) [50]. Participants often sought care from providers who were well-informed of the appropriate healthcare practices and procedures particular to TGD people. However, the lack of access due to limited numbers of gender-affirming providers was declared the most impactful barrier to healthcare among several participants (*n* = 6 of 19 participants with 7 total endorsements). A shortage of knowledgeable gender-affirming providers made it extremely difficult for participants to access appropriate care, which resulted in a diminished quality of care or no care at all as seen in a quote from Ruby, *“I’ve tried but the list [for counseling]*…*the waiting list is*, *impossible at [LGBTQ health center]*. *The list is impossible*, *so*…*yeah*, *I’ll just have to wait*.*”* In addition to lack of access to mental health providers, participants also noted long waiting lists to access gender-affirming surgeons and gender-affirming primary care providers. Moreover, structural healthcare delivery was often not gender-affirming. Healthcare records and health insurance information did not always accurately represent the participant’s gender identity, causing confusion and unintentionally stigmatizing experiences with healthcare providers and staff. For example, Labradorite shared,

*[O]ne time I was inside the lobby waiting for my name to be called to be seen by the doctor. So they would say mister and have my boy name*… *When I got my name legally changed then they say it, mister, coz they still wasn’t looking at the gender marker, so I had to fight for all those different things*.

As seen above, healthcare staff often failed to refer to the participant by their correct name, pronoun, or gender identity due to inconsistencies in documentation. This created tension and discomfort in the healthcare setting.

### Rapport and continuity

Rapport refers to a relationship of trust and mutual understanding between people [51]. Continuity refers to coherent and interconnected healthcare services that are consistent with a patient’s healthcare needs and preferences. Rapport and Continuity encompasses our themes *Stigma and Mistreatment by Healthcare Staff*, *Misalignment of Provider and Patient Perception of Care*, *Complex Social and Structural Vulnerability*, and *Continuity of Care* (see Table 2 for definitions of themes) and corresponds to BHI items: *Previous bad experiences*, *Feel like healthcare providers are not comfortable caring for Transgender patients*, *and Mistreatment by healthcare staff and/or other patients for being transgender*.

#### Stigma and mistreatment by staff

Another commonly endorsed highly impactful barrier to healthcare was stigma and mistreatment by healthcare staff (*n* = 6 of 19 participants with 10 total endorsements). Anticipated stigma often occurred in relation to HIV status. Participants expressed feelings of being scared to seek treatment for HIV out of fear of the diagnosis and fear of being judged. Spectrolite shared that she delayed treatment due to fear of the disease and fear of how she would be treated by healthcare professionals. Spectrolite’s fears lead her to futile feelings such as seen in this quote, *“I got my [HIV] care late ’cause at first I was scared*, *I was like*, *girl*, *I’m a just die*.*”* These fears of discrimination, noted by Spectrolite, were echoed by three other participants.

Interpersonal stigma presented as discrimination and mistreatment from healthcare providers and fellow patients related to gender identity, HIV status, and substance use. Participants often felt they were being judged for showing up at clinics that specialized in HIV care. Participants relayed instances where others were gossiping about or judging them based on their appearance (i.e., skin and weight altered by HIV) or suspected substance use. Sunstone shared that she was unable to get the pain treatment she needed due to stigma related to opioid use and her gender presentation and identity:

*I had a nurse here that made my healing and process that came through cancer horrible and hard because she was afraid I was going to be addicted to opioids… she was giving me a hard time in order for me to get my prescriptions filled and like I had anal cancer. I don’t know…I guess all cancer bad*…*but it was literally…in my butt so that means bathroom time was horrible. She didn’t take accountability that I was like…the pain medicine you’re giving me is not stopping my pain…I will never forgive her*.

*[Also,] …even in a gay clinic it still happens where you’re misgendered or you feel a certain way where you feel the other person is judging or looking at you and everybody doesn*’*t come in one cookie cutter…some of us are bigger, some of us are smaller…I’m a human being…Some people are rude, some people intentionally misgender you it’s like it’s a joke to you*.

As seen above, healthcare providers would assume the participant was using illicit drugs or misusing prescribed pharmaceutical drugs, which may have led to inadequate care and outright accusations and mistrust. Such actions by the healthcare team resulted in shame, isolation, and frustration with inadequate care among participants.

Confidentiality concerns regarding stigmatizing health information (*e*.*g*., HIV status) emerged as a subtheme of *Stigma and Mistreatment by Staff* (*n* = 3 of 19 participants with 4 total endorsements). Participants described instances where their confidentiality was breached or reported fears of a potential breach. Sunstone described the following breach, *“I find that a lot of [hired LGBTQ+ people in the clinic… they get in the position and*… *there’s instant chit chatter*…*it’s in the DNA… ‘Oh you know [Sunstone] came in here*, *she needed help with*… .*’”* As seen in this quote, breaches of confidentiality were often believed to have been caused by LGBTQ+ peers working in clinic settings. However, breaches of confidentiality were not always apparent. Spectrolite shared,

*[I]n the past before this one clinic it was a, a classmate who mom worked at the place. And she end up bein’ there and I didn’t like to go there anymore*. *Well, it was my concerns of the classmate knowin’ my business ’cause the clinic was only for that reason [HIV]*.

This fear of a breach of confidentiality was enough to make participants apprehensive about receiving care in a clinic setting where their LGBTQ peers or known acquaintances worked and delay treatment seeking.

#### Misalignment of provider’s and patient’s perception of care

One of the most impactful barriers to healthcare was when the provider’s perception of care did not match that of the patient (*n* = 5 of 19 participants with 6 total endorsements). This issue occurred during both elective and medically necessary treatments. A participant wanting gender-affirming surgery was denied by a surgeon who felt it was not needed and eventually sought care elsewhere with extended wait times. In another example, Amethyst shared that her provider did not respect her wishes to discontinue her medication for depression,

*I started having a depressed spirit… the doctor put me on medications. And I don’t like a lot of medications… and then one medication was making me feel like a zombie*. *I said ‘No, I don’t need this medication.’ He said ‘You don’t want that?’ I said "No". And I stopped taking that medication [even though the doctor did not change the treatment plan]*.

As seen with Amethyst, other participants often had to make their own alterations to their care, such as medication adherence or continued attendance to counseling sessions, based on misalignments in perceptions of necessary care and failed communication between the provider and the participant. Participants who experienced the provider’s perception of care misaligning with their own felt dismissed or undertreated and were unhappy with their treatment, or lack thereof.

*Complex social and structural vulnerability*. Participants experienced varying degrees of complex social and structural vulnerability as one of the most impactful barriers to healthcare, including food and housing insecurity, lack of adequate income, substance use, and neighborhood disorder (i.e., “clustering of negative physical and social conditions such as violence, housing problems, economic stress, and drug market activity” p. 3) [52] (*n* = 4 of 19 participants with 5 total endorsements). Sunstone described the importance of reducing social and structural vulnerabilities to improve one’s health and healthcare experience, *“[I]f you want your healthcare to be good*, *all the avenues*,*”* described as access to food and safe shelter, “*from housing to the pharmacist to the doctor has to be [addressed] for the patient’s best care*.*”* When basic needs like food and shelter went unmet, participants often reported disrupted care.

Moreover, Sunstone connected decreased health with food insecurity when she shared, “*I didn’t eat*. *I didn’t… get malnutrition but I knew I wasn’t on top of my game because I was just worried where I was going to lay my head…It’s like all these emotions came up with trying to get settled in housing so it does affect your health*.*”* Similarly, Amber experienced disruptions in care related to having primary needs that superseded their healthcare needs, *“I was going to therapy*, *but something be happening*, *and I just worry about more surviving*.*”*

Lastly, while managing substance use disorder, two of 19 participants did not receive adequate concurrent care for other health conditions. For example, Labradorite prioritized her substance use disorder treatment and recovery over seeking HIV treatment: *“I came into [substance use disorder] recovery and I had about four years clean and that’s when I dealt with my HIV status*.*”* While another participant was told that they could not receive care for their mental health symptoms until they stopped using substances. Both avenues often resulted in delayed treatment. Such disruptions in care and decreased health caused by complex social and structural vulnerability highlight the increasing need for quality holistic care among this group.

#### Continuity of care

Participants (*n* = 3 of 19 participants with 5 total endorsements) identified continuity of care as one of the most impactful barriers to healthcare. Some participants had developed strong relationships with their providers before being abruptly switched to new provider due to the providers’ personal circumstances or institutional changes. Others received healthcare services in locations where the providers were different at each appointment or changed periodically over time based on case load. Amber described delaying treatment-seeking due to inconsistent providers, she shared, *“[I] usually go to like*, *um*, *[LGBTQ health care provider for healthcare]*. *[It’s] usually a different person every time I go in*.*”* Inconsistent access to the same provider resulted in participants feeling like their needs were not met, while others felt apprehensive about receiving care from new providers. Some participants did not follow through with care due to continuity of care, as seen with Amber.

### The effect of barriers to accessing healthcare on the association between polyvictimization and PTSD and depressive symptom severity among Black transgender women

#### Moderation

There was a significant interaction between PVI and BHI, suggesting that the association between PVI scores and PTSD symptom severity is moderated by the number of barriers experienced (Table 5). The overall regression model including PVI, BHI, an interaction between PVI and BHI, age, and location, explained 29% of the variance in PTSD symptom severity (*F*(5,150) = 13.13, *p* < .01). In this sample, a one unit increase in PVI (exposure to an additional type of violence) and a one unit increase in BHI (exposure to an additional barrier) were associated with increased PTSD symptom severity (Standardized Beta [b] = .46, p < .001), after controlling for age, location, and the interaction between PVI and BHI (Table 5). No significant moderating effects were noted between PVI, BHI, and depressive symptoms–and were subsequently not reported in detail in this article.

**Table 5:** Variance in symptoms of PTSD uniquely explained by polyvictimization and barriers to healthcare among the quantitative sample (N = 151).

Variables included in the model	*R* ^*2*^	Adj. R^2^	ΔR^2^	b^ (SE b^)	b
Model 1: Age, Location	0.06	0.04	0.06*		
Model 2: Age, Location, PVI	0.18	0.17	0.13***		
Model 3: Age, Location, PVI, BHI	0.28	0.26	0.1***		
Model 4: Age, Location, PVI, BHI, Interaction (PVI X BHI)	0.31	0.29	0.03*		
Age				-.01(.01)	-.12
Location				-.25(.23)	-.08
PVI				.15(.03)***	.46
BHI				.49(.11)***	.69
Interaction (PVI X BHI)				-.03(.01)*	-.49

Note

*(p< 0.05; 2-tailed)

**(p< 0.01; 2-tailed)

***(p< 0.001; 2-tailed); ^ (unstandardized coefficient); b (standardized coefficient); BHI (Barriers to Healthcare Inventory); Location (0 = DC; 1 = Baltimore); PVI (Polyvictimization Inventory).

#### Mediation

Table 6 shows the impact of PVI and BHI on PTSD symptom severity among participants. In step 1, the PVI explained 11.9% of variance in PTSD symptom severity with F(3,147) = 6.6176, p < .001. The findings revealed that PVI positively predicted PTSD symptom severity (b = .3170, P < .001). In step 2, PVI and BHI explained 28.1% variance in PTSD symptom severity with F(4, 146) = 14.2746, p < .001.The findings revealed that PVI (b = .2622, p < .001) and BHI (b = .3357, p < .001) positively predicted PTSD symptom severity. The regression weights for PVI subsequently reduced from Model 1 to Model 2 (.3170 to .2622) but remained significant which confirmed the partial mediation. More specifically, PVI has a direct and indirect effect [Effect = .0338, 95% C.I. (.0156, .0548)] on PTSD symptom severity, when accounting for BHI, age, and location.

**Table 6:** Regression analysis for mediation of barriers to healthcare access between polyvictimization and PTSD symptom severity among the quantitative sample (N = 151).

Variable	b^	(SE b^)	95%CI	b
**Step 1**				
Constant	2.1234**	.6809	(.7777, 3.4690)	
PVI	.1413***	.0356	(.0708, .2117)	.3170***
Age	-.0155	.0133	(-.0418, .0108)	-.0921
Location	-.2448	.3658	(-.9678, .4781)	-.0545
**Step 2**				
Constant	1.0722*	.4538	(.1753, 1.9691)	
PVI	.0832***	.0242	(.0354, .1310)	.2622***
BHI	.2390***	.0532	(.1338, .3442)	.3357***
Age	-.0134	.0086	(-.0305, .0037)	-.1117
Location	-.2821	.2365	(-7495, 1853)	-.0882

Note

*(p< 0.05; 2-tailed)

**(p< 0.01; 2-tailed)

***(p< 0.001; 2-tailed); ^ (unstandardized coefficient); b (standardized coefficient); BHI (Barriers to Healthcare Inventory); CI (confidence interval); Location (0 = DC; 1 = Baltimore); PVI (Polyvictimization Inventory).

Table 7 shows the impact of PVI and BHI on depressive symptom severity among participants. In step 1 the PVI explained 11.9% of variance in depressive symptom severity with F(3,147) = 6.6176, p < .001. The findings revealed that PVI positively predicted depressive symptom severity (b = .3170, P < .001). In step 2 PVI and BHI explained 13.9% of variance in depressive symptom severity with F(4, 146) = 5.8867, p < .001. The findings revealed that PVI (b = .1386) and BHI (b = .2399, p < .01) positively predicted depressive symptom severity. The regression weights for PVI subsequently reduced from model 1 to Model 2 (.3170 to .1386) but did not remain significant which confirmed the full mediation. More specifically, the indirect effect of PVI on depression symptoms was found to be statistically significant [Effect = .0275, 95% C.I. (.0076, .0519)].

**Table 7:** Regression analysis for mediation of barriers to healthcare access between polyvictimization and depressive symptom severity among the quantitative sample (N = 151).

Variable	b^	(SE b^)	95%CI	b
**Step 1**				
Constant	2.1234**	.6809	(.7777, 3.4690)	
PVI	.1413***	.0356	(.0708, .2117)	.3170***
Age	-.0155	.0133	(-.0418, .0108)	-.0921
Location	-.2448	.3658	(-.9678, .4781)	-.0545
**Step 2**				
Constant	1.4707*	.5650	(.3541, 2.5873)	
PVI	.0501	.0301	(-.0095, .1096)	.1386
BHI	.1944**	.0663	(.0634, .3253)	.2399**
Age	-.0096	.0107	(-.0309, .0116)	-.0705
Location	-.4998	.2944	(-1.816, .0820)	-.1374

Note

*(p< 0.05; 2-tailed)

**(p< 0.01; 2-tailed)

***(p< 0.001; 2-tailed); BHI (Barriers to Healthcare Inventory); CI (confidence interval); Location (0 = DC; 1 = Baltimore); PVI (Polyvictimization Inventory).

## Discussion

Our results confirm previous evidence that Black transgender women experience high polyvictimization and associated mental health symptoms [10, 11]. Findings suggest that access to high quality and competent healthcare services may be instrumental in modifying the association between polyvictimization and PTSD and depressive symptom severity among Black transgender women. However, Black transgender women face a multitude of obstacles to accessing the care they require [8, 53, 54]. Among our sample, 77% of quantitative participants (N = 151) and 84% of qualitative participants (N = 19) reported encountering at least one barrier to healthcare. While a number of the barriers reported by study participants are common to the general population in the US [55–57], some barriers specific to Black transgender women did emerge. Joint display in Table 2 illuminated three domains to describe how barriers to healthcare present among Black transgender women–*Affordability*, *Accessibility*, and *Rapport and Continuity*. Importantly, findings from the multivariate linear regression modeling and pathway analysis suggest that the association between the number of types of violent experiences and PTSD and depressive symptom severity may depend on the number of healthcare barriers experienced. Therefore, understanding and addressing healthcare barriers for Black transgender women is important for identification of actionable, effective interventions.

When discussing these findings, it is important to understand the historical and present-day context in which these participants live. The cities of Baltimore, MD and Washington, DC have a known history of discrimination in lending practices (i.e., redlining) resulting in residential segregation and unequal allocation of resources [58]. Residence can influence healthcare service delivery and access. This pattern persists today as Black people are disproportionately living in areas of lower socioeconomic status [58], resulting in poorer access to healthcare [59]. This phenomenon was also highlighted by our CAB members who emphasized the contribution of one’s social disadvantages on their overall perceived access to and engagement in healthcare. When social factors, including residence, and healthcare access are equal, disparities in accessing healthcare services are reduced [60]. When exploring relationships between social positioning, residence, and health-related outcomes, tenets of intersectionality require use of a historical lens to examine the complex interplay between experiences of privilege and oppression and how they affect one’s outcomes [44, 61–63]. Thus, a discussion of historical and present discriminatory practices will be used to inform our findings regarding healthcare access and engagement of Black transgender women from Baltimore, MD and Washington, DC.

### Barriers to healthcare modify the association between polyvictimization and PTSD and depressive symptom severity

Study participants who experienced one or more barriers to healthcare had significantly worse PTSD and depressive symptom severity. While BHI partially moderated and partially mediated the relationship between polyvictimization and PTSD symptom severity, BHI fully mediated the relationship between polyvictimization and depressive symptom severity among participants. This highlights a significant mental health morbidity resulting from impaired access to healthcare services. Similar patterns have been seen among TGD adolescents experiencing barriers to gender affirming health services [64]. It is of paramount importance that barriers to high quality, effective, and respectful healthcare affecting this community are remedied.

Many prevalent barriers endorsed on the BHI were not identified as one of the most impactful barriers qualitatively. For instance, ‘Mistreatment by Healthcare Staff’ was the least endorsed on the BHI; however, *Experiences of Stigma and Mistreatment by Healthcare Staff* was reported as the most impactful barrier in the qualitative study. Thus, the most common barrier might not be the most impactful one. Similarly, ‘Transportation’, ‘Time’, and ‘Cost’ were commonly identified in the quantitative survey, whereas qualitative study participants reported *Lack of Access to Gender-affirming Care*, S*tigma and Mistreatment by Healthcare Staff*, and *Misalignment of Provider and Patient Perception of Care*, as the most impactful barriers to accessing and engaging in care.

Lastly, several of the most impactful barriers identified by participants were not captured in the quantitative barriers to accessing healthcare measure (BHI), including the *Lack of Access to Gender-affirming Care*, *Misalignment of Provider and Patient Perception of Care*, *Continuity of Care*, and *Complex Social and Structural Vulnerability*. This misalignment may be due in part to some of the most impactful barriers being related to engagement in healthcare compared to accessing healthcare (Table 2). Overall, the barriers reported by our study participants were categorized into three overarching domains, (1) affordability, (2) accessibility, and (3) rapport and continuity, and are discussed below.

### Affordability

Financial barriers to accessing healthcare in the US have long been a leading point of concern with insurance status being the most significant determining factor [56]. Lack of insurance, concerns for financial strain, and concerns for cost were all cited as significant barriers to healthcare in this sample. However, social desirability or other sources of information bias could have affected the study findings. A cost analysis has concluded that coverage of gender- affirming care–hormone replacement therapy, gender affirming surgery, primary care services, and mental health services–is affordable at approximately $0.016 per member per month, and cost-effective compared to treating the negative consequences of denial of care–HIV, depression, suicidality, drug abuse, etc. [65]. While the introduction of the Affordable Care Act (ACA) in 2010 effectively expanded coverage to over 19 million Americans including gender, sexual, and racial minorities, subsequent actions to limit the ACA’s reach have resulted in a rebound in uninsured rates [66]. These decisions have a direct impact on the most vulnerable members of society including Black transgender women. Legislation, such as the Equality Act, which provides provisions against discrimination related to gender identity has been opposed by the Trump administration [67]. In removing these protections for Black TGD people, job discrimination can limit people’s access to health insurance and income [67]. Approximately, 8.5% of the general US population were uninsured in 2018 [68], however, 15% percent of quantitative participants specifically named lack of insurance as a barrier to healthcare. Sixteen percent of qualitative participants reported a lack of adequate insurance, as well as dependency on limited government or charity payouts, as a barrier to accessing and engaging in healthcare. Policy makers and lobbyists need to push back on limitations to LGBTQ protections under the law; they also need to fight for access to insurance and full coverage of all medically-necessary gender-affirming care [69].

### Accessibility

Transportation, time, inconvenient operating hours, and fears for safety getting to and from the health setting all emerged as barriers to accessing care among quantitative participants. Transportation was the most highly endorsed barrier in the study which aligns with experiences in the general population. It is estimated that 5.8 million insured adults in the general US population miss or delay medical treatment every year secondary to challenges with transportation [57]. Transportation barriers are, therefore, even more salient for those with lower incomes or those who are underinsured or uninsured [57, 69–71], which is the case for a significant proportion of our study participants. Our study sample was made up entirely of Black transgender women; African Americans, in particular, have the highest burden or challenges associated with transportation to care, relying heavily on outdated and poorly designed public transportation systems [57, 72].

In national samples, African American people are less likely than other racial subgroups to attend follow-up appointments, often related to community disparities in transportation [60]. Despite being a model city for a public transit system, Baltimore’s system has failed to effectively connect urban residents to a means of work. The failure of this once promising system of public transportation, has been attributed to White flight, suburbanization, and a dependence on automobiles [73]. As a result, urban residents, who depend on the public transport system, are subjected to waiting hours for buses that frequently break down or are too full to pick up more riders [73]. These failures in public transport are likely to be exacerbated as transit systems are further limited in response to the COVID-19 pandemic [74]. These structural changes in transportation further inhibit low-income African American residents (without automobiles) by requiring longer, indirect public transportation routes, resulting in additional time for transportation to and from clinics that must be coordinated within the clinic’s hours of operation [73]. Doing this could require time away from work which may not be feasible and may introduce additional barriers in terms of ability to afford care.

The use of public transport to and from healthcare visits can be very unsafe for Black transgender women who risk stigma fueled harassment and assault, which adds an additional layer of complexity to accessing and engaging in healthcare services [24, 75]. Research conducted on the public transit system in Portland, Oregon highlighted the fear of harassment, discrimination, and violence experienced by transgender people that led to an additional toll of “immobility” [75]. Immobility was described as limiting the number of trips taken or avoiding certain transit stops [75]. When we consider the residential context of our findings, safety beyond the experiences with the transit system emerge.

Racism and cisgenderism, ingrained in every system within the US, limit the socioeconomic opportunities of Black people in general and Black transgender people in particular. The media portrays both Black and transgender bodies as less valuable, further engendering compliance with white supremacy and cisgenderism [76]. Consequently, Black people are disproportionately arrested and incarcerated. This coupled with the fact that Black transgender women report high rates of rejection by biological family members, severely reduces their access to familial social support and social resources needed to manage discrimination [77]. Racist and cisgenderist policies act to limit the access of Black families and Black transgender women to resources and inhibits their ability to maintain the health and wellbeing of their households. In return, poverty and income inequalities have been directly associated with inhibited transportation to and from healthcare services [78].

### Rapport and continuity

Among our qualitative participants issues of competence, respectful treatment, consistent access to the same provider, and confidentiality all emerged as the most significant barriers to access and engagement in healthcare. In addition to usual care, transgender patients often require additional interventions such as hormone therapy, surgery, and management of complex social vulnerabilities (*e*.*g*., home, income, and food insecurity); which require specially trained interdisciplinary teams of providers. With our qualitative participants reporting lack of access to gender-affirming care, our findings align with others that have found the single largest barrier to engaging in care among transgender adults is a lack of gender-affirming care from well informed, competent providers with expertise in TGD medicine [79, 80]. While this was not the most widely endorsed barrier to accessing care among our quantitative participants, many endorsed feeling like healthcare providers were not comfortable taking care of transgender patients as a barrier to accessing care. This finding supports previous research focused on healthcare encounters from the perspectives of transgender patients and healthcare providers [81]. Transgender patients describe the encounters as being mutually discomforting by subjecting their care to providers who were uncomfortable with providing care or even acknowledging the existence of transgender patients [81]. Healthcare providers’ lack of comfort with transgender patients is not surprising given the lack of didactic and clinical exposure during clinical education but should be rectified through the integration of transgender health in related medical topics [82, 83]. Education for all healthcare providers must include training in the treatment and management of the basic health needs of TGD people.

Inexperienced healthcare providers only represent a portion of the problem. Even when our study participants sought care at LGBTQ+ focused specialty clinics, they reported being disrespected, stigmatized, gossiped about and otherwise mistreated by clinic staff or other patients. Quantitative participants reported “previous bad experiences” in general were a significant barrier to accessing healthcare. Our study confirms findings that most negative healthcare experiences for Black transgender women are secondary to poor communication [84]. Specifically, qualitative participants reported concerns with confidentiality breeches perpetrated, most commonly, by LGBTQ+ peers working in the clinic setting. Breaches of confidentiality can have serious and sometimes life-threatening consequences for TGD people particularly in the setting of an involuntary “outing” [85]. Participants expressed difficulty getting the care they requested when providers did not perceive their desired care as necessary (*e*.*g*., gender-affirming cosmetic procedures) leaving them feeling unheard and not respected while also creating an additional healthcare barrier by forcing them to seek these services elsewhere. Safe and trusting relationships with the healthcare team is a key factor encouraging ongoing engagement in care for Black transgender women [84]. Efforts should focus on interventions designed to decrease TGD-related stigma and discrimination in the healthcare setting.

Another significant barrier to access and engagement in care that emerged from the qualitative interviews was a lack of continuity with healthcare providers. Low variability in healthcare teams is essential for the establishment of trust and familiarity between a patient and their providers [86]. This trust fosters a relationship that potentially leads to increased quality of care, increased engagement in care, and ultimately, improved health outcomes [86]. Institutional changes and provider turn-over meant that study participants frequently bounced between different providers making it more difficult for participants to get their needs met. It also created a sense of loss, especially in circumstances when study participants had already developed a strong relationship with a particular provider. Qualitative participants reported delaying or avoiding care secondary to issues with continuity.

### Limitations

A number of limitations should be considered in the interpretation of the study findings, including the modest sample size and lack of co-variables (e.g., having a primary care provider, availability of health insurance) in the multivariate models. In addition, the cross-sectional study design prohibits interpretation of our results as causal. The community-based purposive sampling method (focusing on Black transgender women in Baltimore, MD and Washington, DC) reduces the applicability of our findings to other transgender communities outside of Black transgender women living in metropolitan cities. Moreover, the experiences of Black non-binary people assigned male at birth were not captured in this study. This decision was made because Black transgender women report experiencing a higher rate of violence compared to Black non-binary people [1, 9, 18]; thus, we were focusing on the community of greatest need. The gender identity data was also limited, because participants were not able to select multiple terms to describe their gender. Thus, people who may identify as a non-binary transgender woman were unable to be recognized. However, the narratives of Black non-binary people are greatly underrepresented in the literature and future research should focus on this community. The quantitative assessment was designed to capture barriers to accessing care, whereas the qualitative interviews captured barriers to both accessing and engaging in care, this nuance may account for differences in findings between the groups. Future research should account for barriers to both access and engagement in healthcare to comprehensively inform intervention development for Black transgender women.

## Conclusion

The convergent mixed-methods approach taken in this secondary data analysis allowed for a unique synthesis of existing data regarding barriers to access and engagement in care and their association with polyvictimization and mental health symptom severity among Black transgender women living in Washington, DC and Baltimore, MD. The insights gained from this analysis present significant implications for practice and future research. Our study found that BHI partially moderated and partially mediated the relationship between polyvictimization and PTSD symptom severity. BHI fully mediated the relationship between polyvictimization and depressive symptom severity among participants. This highlights a significant mental health morbidity resulting from impaired access to healthcare services. Although many of the barriers reported by study participants are common to the general US population, some barriers unique to Black transgender women did emerge. Improving access to healthcare may mitigate the association between polyvictimization and mental health symptoms among Black transgender women. Future research should focus on interventions designed to alleviate barriers to access and engagement in health care in the vulnerable population.

## Supporting information

S1 Table Acronyms. (DOCX)
