# Supplementary material for: Barriers to accessing and engaging in healthcare as potential modifiers in the association between polyvictimization and mental health among Black transgender women
Source: PLoS One. 2022 Jun 16;17(6):e0269776. doi: 10.1371/journal.pone.0269776 (PMC9202936; doi:10.1371/journal.pone.0269776)
Supplement: S1 Table — (DOCX) [file pone.0269776.s001.docx]

| **S1 Table. Acronyms** | |
| --- | --- |
| **Acronym** | **Meaning** |
| BHI | Barriers to Accessing Healthcare Inventory |
| PTSD | Post-traumatic Stress Disorder |
| TGD | Transgender and Gender Diverse |
| USTS | United States Transgender Survey |
| PVI | The Polyvictimization Inventory |
| PHQ2 | The Patient Health Questionnaire 2 |
| PC-PTSD | The Primary Care Post Traumatic Stress Disorder Clinical Screener |
| HIV | Human Immunodeficiency Virus |
| LGBTQ | Lesbian, Gay, Bisexual, Transgender, and Queer |
